# EF Bart's Disease with Coinheritance of G*γ*-XmnI and A*γ*-Globin Polymorphisms: A Case of Nontransfusion-Dependant Thalassemia

**DOI:** 10.1155/2020/8869335

**Published:** 2020-10-30

**Authors:** Kane M. Laks, Cara Hirner, Barbara Gruner, Jared Coberly, Katsiaryna Laziuk, Bindu Kanathezhath Sathi

**Affiliations:** ^1^Department of Pediatrics, University of Missouri School of Medicine, Columbia, MO, USA; ^2^Department of Pathological Sciences, University of Missouri School of Medicine, Columbia, MO, USA; ^3^Pediatric Hematology Oncology, Valley Children's Hospital, Madera, CA, USA

## Abstract

EF Bart's disease is a rare form of nontransfusion-dependant thalassemia (NTDT) due to the coinheritance of homozygous hemoglobin *E* (*β*^E^/*β*^E^) genotype with hemoglobin H disease. These individuals are routinely found to have thalassemia intermedia with moderate anemia, increased hemoglobin Bart's and hemoglobin F on electrophoresis. The contribution of hemoglobin F-inducing polymorphisms in this disease has not been described previously. Here, we describe the hematological profile in a young child with coinheritance of G*γ*-XmnI and A*γ*-globin gene polymorphisms in EF Bart's disease. Interestingly, in this rare form of NTDT, normal HbF and elevated HbA2 were noted.

## 1. Introduction

The hemoglobin (Hb) tetramer consists of 2 *α*-globin chains and 2 *β*-globin chains. *β*-Thalassemia results from reduced (*β*^+^) or absent (*β*^0^) synthesis of the *β*-globin chains, with 3 possible phenotypes consisting of clinically asymptomatic *β*-thalassemia carriers (heterozygotes), transfusion-dependent anemia in thalassemia major, and a wide range of genotypes and clinical features of nontransfusion-dependant thalassemia (NTDT) [1, 2]. The *aα*-thalassemia consists of *α*^+^-thalassemias, with only one of the linked pair of *aα* genes deleted (–*α*/*αα*), and *α*^0^-thalassemias, with both genes deleted (–/*αα*) [3–5]. While *α*^+^-thalassemia heterozygotes are clinically asymptomatic, *α*^0^-thalassemia heterozygotes and *α*^+^-thalassemia homozygotes exhibit *aα*-thalassemia trait, with similar features as *β*-thalassemia trait. HbH disease results from a 3-gene deletion, occurring when both *α*^+^-thalassemia and *α*^0^-thalassemia (–*α*/--) are inherited [6, 7]. HbE is a mildly unstable *β*-hemoglobin variant, with heterozygotes displaying minimal morphological red blood cell abnormalities and with homozygotes exhibiting significant morphological abnormalities and mild anemia, similar to heterozygous *β*-thalassemia. HbE commonly co-occurs with forms of *β*-thalassemia and *aα*-thalassemia, given that all 3 hemoglobinopathies are common in Asia [8–10].

EF Bart's disease (HbH/EE) results from interaction of *aα*-thalassemia (--/-*α* or --/*α*^cs^*α*) mutation with homozygous HbEE (*β*^E^/*β*^E^) or (*β*^E^/*β*^0^), with a clinical phenotype of thalassemia intermedia [10]. The hemoglobin profile consists of HbE and often highly elevated levels of HbF, with the remainder being Hb Bart's [11]. Phenotype consists of mild-to-moderate anemia requiring intermittent transfusion therapy, similar to HbH disease [10]. The cases of EF Bart's disease reported to date have not been examined for the presence of the A*γ*-globin gene polymorphism, recently discovered and shown in *β*-thalassemia syndromes to activate the A*γ*-globin gene, with a consequent induction of HbF expression manifesting as a milder phenotype and lower likelihood of requiring transfusions [12]. Frequently found to co-occur with the A*γ*-globin gene polymorphism is the XmnI polymorphism which enhances HbF during the mild but chronic erythropoietic stress exhibited in carriers of *β*-thalassemia [12, 13]. Although these polymorphisms have been shown to induce HbF in *β*^0^-thalassemia syndromes, their contribution in *aα*-thalassemia syndromes, or complex heterozygous thalassemia syndromes, is relatively unknown [12, 14, 15]. Here, we describe coexistent A*γ*-globin *G* > *A* polymorphism and G*γ*-XmnI polymorphism in the setting of EF Bart's disease resulting in nontransfusion-dependant thalassemia with elevated HbA2.

## 2. Methods

This case report was approved by the institutional review board of University of Missouri, Columbia, MO, USA. Parental consent was obtained for this report. *α*-Gene sequencing was conducted by direct mutation analysis, with *α*-globin locus deletions and Hb Constant Spring point mutations being identified by multiplex ligation-dependent probe amplification assay. In order to map the approximate location of DNA deletions, fifteen probes hybridizing throughout the *α*-globin locus were utilized, from the HS40 promoter region through the 3′HVR region. A PCR-based assay was also used, detecting the presence of *α*−3.7 and *α*−4.2 deletion [16].

Steps of *β*-gene sequencing began with the extraction of genomic deoxyribonucleic acid (DNA) from whole blood, followed by the amplification of HBB gene by polymerase chain reaction (PCR), then purification, and sequencing of the PCR product in both directions with fluorescent dye-terminator chemistry. An automated sequencer was used to separate sequencing products, with trace files being analyzed for variations in all exons and introns other than IVS-II-82 through IVS-II-650, 5′untranslated region (UTR), 3′UTR, and the promoter region. Routine studies were then correlated with the results to identify unusual *β*-globin variants [17].

The *γ*-globin full gene sequencing consisted of total genomic DNA being extracted from the sample, followed by full *γ*-globin genes being amplified by PCR in separate reactions, and then Sanger sequencing. A combination of automated calls and manual inspection was used to review the sequence data (test code: WGSEQ, Mayo Laboratories) [18].

## 3. Results

**Figure 1:**
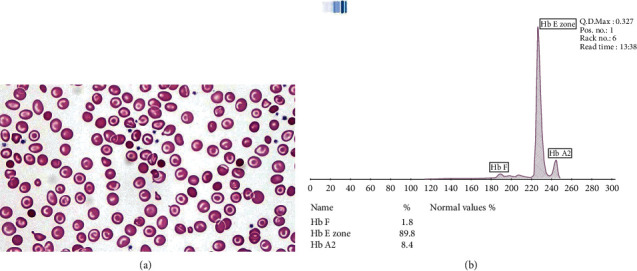
(a) Photomicrographs of Wright-stained peripheral blood film showing microcytosis and hypochromasia. There are multiple-target red blood cells and rare spherocytes and teardrop cells (Wright stain, 1000x). (b) Capillary hemoglobin electrophoresis pattern demonstrating homozygote hemoglobin E (HbE) variant and elevated hemoglobin A2 (HbA2) with the absence of hemoglobin A1 (HbA1) in the peripheral blood sample.

The index case was a 2-year-old female of Filipino descent who presented to our hematology clinic with microcytic anemia. Growth parameters and iron indices were normal in this child. Both sister and brother of the index case were previously treated at our clinic for anemia occurring from thalassemia (Table 1). The child had mild hepatosplenomegaly, and there was no evidence of thalassemic facies. Hematological indices (Table 1), peripheral blood smear (Figure 1(a)), and Hb electrophoresis (Figure 1(b)) were obtained.

A strong family history of *α*-thalassemia and HbE disease was present (Table 1). The mother had *α*-thalassemia trait with 2 *α*-globin gene deletion in the cis position, Southeast Asian (SEA) variant (*α* genotype, ^SEA^/*αα*), and HbE trait (*β* genotype, *β*/*β*^E^), with microcytic anemia. The father had *α*-thalassemia trait with a single *α*-globin gene deletion and heterozygosity for Hb Constant Spring (CS) (*α* genotype, *α*^3.7^/*α*^CS^*α*), as well as HbE trait (*β* genotype, *β*/*β*^E^), with microcytic anemia. The brother had HbH disease with 2 *α*-globin gene deletion in the cis position, SEA variant, and an *α*-gene deletion on the trans allele (*α* genotype, ^SEA^/-*α*^3.7^, and *β* genotype, *β*/*β*). The sister was a compound heterozygote with *α* genotype, −^SEA^/*α*^CS^*α*, and *β* genotype, *β*/*β*^E^ (HbH-CS/*E* trait).

### 3.1. Genotyping Results

*α*-Gene sequencing revealed 3 *α*-genes deleted, a single *α*-gene in the −3.7 kb on one chromosome, and 2 genes in the other chromosome (*α* genotype−^SEA^/−*α*^3.7^).

*β*-Gene sequencing revealed HBB had *β* 26, GAG > AAG, Glu > Lys, resulting in homozygous *E* mutation (*β* genotype, *β*^E^/*β*^E^).

*γ*-Globin full gene sequencing revealed that, in chromosome 11, HBG1 A gamma, 5′ UTR + 25, *G* > *A*, was identified in heterozygous status with resultant A*γ* (*G* > *A*) polymorphism. Additionally, in the gene HBG2, G*γ*, promoter-158, *C* > *T* polymorphism was detected with heterozygous state for XmnI polymorphism. DNA sequencing did not reveal the presence of these polymorphisms in the brother or sister.

## 4. Discussion

HbE/*β*-thalassemia with *α*^0^-thalassemia/*α*^+^-thalassemia (HbH disease) results in NTDT thalassemia syndrome called EF Bart's disease [11]. This disease is usually characterized by hypochromic microcytic red blood cells with variable severities, but is generally milder than homozygous *β*^0^-thalassemia. Although these patients are not routinely transfusion-dependent, they may need intermittent transfusion during episodes of increased hemolysis triggered by infection or conditions of increased oxidative.

Stress [19] red cell transfusion therapy may also be required during periods of severe anemia, pregnancy, growth delay, exercise intolerance, and poor school performance [19].

The hemoglobin range in EF Bart's disease has been described to be around 7.4 ± 1.6 g/dL, but our patient has consistently shown a higher steady-state hemoglobin levels than previously reported in the literature [11, 20].

Further complexity can be added to the clinical picture of compound heterozygous thalassemia syndromes by inducers of HbF, which are often protective. For example, the recently described A*γ*-globin gene polymorphism, a *G* > *A* polymorphism present at position +25 of the A*γ*-globin genes, was detected in our case. This is part of a sequence recognized by DNA-binding protein complexes including LYAR (Ly-1 antibody reactive clone), a transcription factor that binds the *γ*-globin gene and silences it, repressing HbF expression. Conversely, the A*γ*-globin gene polymorphism alters the binding activity of LYAR and activates the A*γ*-globin gene, inducing HbF expression with a milder phenotype and lower likelihood of requiring transfusions [12, 20]. This polymorphism and the resultant increase in HbF levels are thus considered to be protective in thalassemia syndromes.

Also detected in our case is the XmnI polymorphism, the most common polymorphism associated with HbF induction [21, 22]. Presence of this polymorphism enhances expression of HbF during erythropoietic stress, notably including the mild but chronic erythropoietic stress exhibited in carriers of *β*-thalassemia, who are thus protected when they express these point mutations [13]. Although heterozygous in our case, the presence of homozygous G*γ*-XmnI polymorphism has been reported in another patient with Hb Lepore-EF Bart's disease, where the patient was found to have a milder phenotype of disease [20]. There is very little compensatory HbF increase in these patients. Also, HbH is usually not detectable due to the excessive *β*^E^_4_ globin chains, which do not form tetramers. Previous studies have shown that the A*γ*-globin gene polymorphism frequently occurs in complete linkage disequilibrium with the G*γ*-XmnI mutation in *β*-thalassemia, resulting in these two polymorphisms known to increase HbF production occurring together [12].

HbE is the major hemoglobin detected in EF Bart's, with variable levels of Hb Bart's and HbF. Elevated Hb Bart's was detectable on routine hemoglobin electrophoresis in our patient, while HbF was within normal limits. Comigration of HbE to HbA2 zone in routine high-performance liquid chromatography can pose a problem in these cases. We were able to quantify HbE and HbA2 levels via capillary electrophoresis, avoiding the comigration that occurs in cellulose acetate agar and citrate agar electrophoresis. Our patient's HbE was highly elevated at 90%, and interestingly, unlike the cases previously reported with EF Bart's disease, this child showed an elevated HbA2 which was the second dominant hemoglobin and normal levels of HbF for age [11]. A diagnostic dilemma was present due to the abnormal hemoglobin electrophoretic pattern in this child, and detailed DNA sequencing analysis was required to confirm the diagnosis.

Compared to EF Bart's disease or Constant Spring EE Bart's disease, our patient showed a milder phenotype with mild anemia. Contrary to the previous report of 2 patients from Thailand, there was no elevation in HbF in this patient [11]. The mild anemia noted in our child could be due to the coexistence of both *α*-thalassemia and homozygous HbEE (*β*^E^/*β*^E^) disease which will result in restoration of some degree balance between the *α-* and *β*-globin chains.

The *α*-globin/non-*α*-globin mRNA ratio has been demonstrated to be a good indicator of disease severity in most of the thalassemia syndromes [23]. The *α*-globin/non-*α*-globin mRNA ratio is widely variable in *β*-thalassemia/HbE patients, with no consistent difference between mild and severe phenotypes [24]. HbE is the result of *G* to *A* mutation in codon 26 of the HBB gene which leads to activation of a cryptic 5′ splice site in codon 25 leading to a reduction in correctly spliced *β*^E^-globin mRNA with resultant *β*^+^ thalassemia [24]. One of the factors that correlate with clinical severity has been found to be the ratio between correctly and aberrantly spliced *β*^E^ mRNA in *β*-thalassemia/HbE disease [24]. The level of mRNA transcript alone will not explain the clinical severity. Other factors involved in transcription, translation, and posttranslational modification of the globin chain, proteolytic capacity of red blood cells, and cellular levels of HbF, ultimately, contributes to the severity of hemoglobin *E* disease [25].

Coinheritance of *α*^0^-thalassemia elevates HbA2 level in homozygous hemoglobin *E* disease [26]. A significant higher expression of Hb A2 has been noted in Hb EE/*α*^0^-thalassemia compared to Hb EE without coexistent *α*^0^-thalassemia. This is likely due to more effective binding of *δ*-globin chain to *α-*globin chain to form Hb A2 (*α*_2_*δ*_2_) in conditions of limited availability of *α*-globin chains as in *α*^0^-thalassemia [26]. The relative elevation in HbA2 in this child could also be due to the survival advantage of the red blood cells with higher *δ* expression in the presence of abnormal *β*^E^ tetramers [25]. Thus, elevation in HbA2 has been found to be a useful marker to differentiate hemoglobin EE disease with and without alpha thalassemia [26].

The interaction of A*γ*-globin gene and G*γ*-XmnI polymorphisms in complex heterozygous hemoglobinopathies is not fully known, but we speculate this to be protective based on the clinical severity of the disease in this child. Unlike the cases described previously [11], there was no evidence of Hb Bart's in this patient as she grew older, indicating reduced formation of *γ*_4_ tetramers [10, 11].

## 5. Conclusion

Our patient is 3 years of age and has been transfusion independent to date. This case also presented us with diagnostic challenge due to abnormal hemoglobin electrophoretic pattern, and the variant haemoglobins were confirmed on capillary electrophoresis. Detailed molecular analysis is required in these cases to delineate the complete genotype. Current management of these individuals is supportive with only intermittent need for transfusion. Splenectomy is not routinely indicated or recommended in these patients. The role of erythroid maturation agents, such as luspatercept or sotatercept [19], in complete amelioration of anemia is unknown at this time and must be undertaken only on a clinical trial basis in these complex heterozygous hemoglobinopathies [27].

## Figures and Tables

**Table 1:** Steady-state hematological indices of index case and family members.

	Proband					Brother	Sister	Mother	Father
Age	1 mo	3 mo	1 yr	2 yr	3 yr				
*α*-Genotype	−^SEA^/−*α*^3.7^					−^SEA^/−*α*^3.7^	−^SEA^/*α*^CS^*α*	−^SEA^/*αα*	−*α*^3.7^/*α*^CS^*α*
*β*-Genotype	*β* ^E/^ *β* ^E^					*β*/*β*	*β*/*β*^E^	*β*/*β*^E^	*β*/*β*^E^
Hb (g/dl)	10.1	8.1	8.9	9.2	9.4	9.1	7.4	12.3	11.5
RBC (10^12^/l)	4.60	5.04	6.29	6.40	6.56	5.91	5.10	6.16	5.12
Hct (%)	32.5	24.6	25.6	28.3	29.0	30.9	27.7	39.1	36.7
MCV (fl)	70.7	48.8	40.7	44.2	44.2	52.3	54.3	63.5	71.7
MCH (pg)	22.0	16.1	14.1	14.4	14.3	15.4	14.5	20.0	22.5
MCHC (g/dl)	31.1	32.9	34.8	32.5	32.4	29.4	26.7	31.5	31.3
Retic (%)	1.11		0.38	0.72	0.37	1.47	7.50	0.97	1.30
HbF (%)	64.1	47	7.6	2.2	1.5	0.0	0.0	0.0	0.0
HbA2 (%)		3.3		8.0	8.0	1.4	2.3	3.7	2.8
HbE (%)		43.3		89.7	90.5	0.0	12.6	17.2	17.2
Hb Bart's (%)		6.4				0.0	0.6	0.0	0.0
HbH (%)						4.4			
HbCS (%)							2.7		
S. iron (mcg/dl)							102		
S. ferritin (ng/ml)					83.3	62.7	498		

Abbreviations: Hb = hemoglobin; Hb CS = hemoglobin Constant Spring; RBC = red blood cell; Hct = hematocrit; MCV = mean corpuscular volume; MCH = mean corpuscular hemoglobin; MCHC = mean corpuscular hemoglobin concentration; retic = reticulocyte; mo = month; yr = year.

## Data Availability

The data used to support the findings of this study are available from the corresponding author upon request.
